# Supplementary material for: Content-rich biological network constructed by mining PubMed abstracts
Source: BMC Bioinformatics. 2004 Oct 8;5:147. doi: 10.1186/1471-2105-5-147 (PMC528731; doi:10.1186/1471-2105-5-147)
Supplement: Additional File 2 — The original results of the above study (non-essential files are deleted to keep the file size under the limit set by BMC bioinformatics). [file 1471-2105-5-147-S2.bz2 › chilibotAdditionalFile2/dip05/373ID7680645E8296/html/FGF1_FGFR4.html]

 


 **FGF1** and **FGFR4** 
  
Found 127 abstracts in PubMed, retrieved 05.  
 

 What does Google say? 
 PDF only 
| .edu only 

---

**Interactive relationship** (e.g. stimulation, inhibition, etc)

**Neutral relationship**- **FGF1** , FGF7, FGF9, FGF10, FGF11, and FGF18 were also expressed in the pancreas at varying time points and levels, as well as FGF  [ **FGF1** ]  receptor forms FGFR1b, FGFR1c, FGFR2b, FGFR2c, FGFR3b, and  **FGFR4** .  Ref: 12666204 Dev Dyn, 2003

**Non-interactive relationship** (e.g. studied together, co-existance, homology, etc.)

- This provides an initial description of the expression in the developing skull of the genes encoding the secreted molecules BMP 2, BMP 4, BMP 7, the transmembrane FGF  [ **FGF1** ]  receptors FGFR 1, FGFR 2, FGFR 4  [ **FGFR4** ] , the transcription factors Msx1, Msx2, and Twist, as well as Goosecoid and the early membranous bone differentiation marker osteopontin.  Ref: 12710966 Dev Biol, 2003
- We demonstrate that FXR directly regulates expression of fibroblast growth factor 19 FGF  [ **FGF1** ]  19, a secreted growth factor that signals through the  **FGFR4**  cell surface receptor tyrosine kinase.  Ref: 12815072 Genes Dev< MedlineTA>Genes Dev, 2003
